# Recent Developments in Using *Drosophila* as a Model for Human Genetic Disease

**DOI:** 10.3390/ijms19072041

**Published:** 2018-07-13

**Authors:** Christine Oriel, Paul Lasko

**Affiliations:** 1 Maternal Infant Child Youth and Research Network, V2-230, 950 West 28th Ave, Vancouver, BC V5Z 4H4, Canada; christine.oriel@cw.bc.ca; 2 Department of Biology, McGill University, 3649 Promenade Sir-William-Osler, Montreal, QC H3G 0B1, Canada

**Keywords:** rare Mendelian diseases, animal models, targeted funding opportunities

## Abstract

Many insights into human disease have been built on experimental results in *Drosophila*, and research in fruit flies is often justified on the basis of its predictive value for questions related to human health. Additionally, there is now a growing recognition of the value of *Drosophila* for the study of rare human genetic diseases, either as a means of validating the causative nature of a candidate genetic variant found in patients, or as a means of obtaining functional information about a novel disease-linked gene when there is little known about it. For these reasons, funders in the US, Europe, and Canada have launched targeted programs to link human geneticists working on discovering new rare disease loci with researchers who work on the counterpart genes in *Drosophila* and other model organisms. Several of these initiatives are described here, as are a number of output publications that validate this new approach.

## 1. Introduction

Efforts to develop the fruit fly *Drosophila* as a model for human disease extend back to the earliest days of fly research. Working in collaboration with Thomas Hunt Morgan and Calvin Bridges exactly a century ago, Mary Stark first described a genetic mutant in flies that causes lethal tumors, thus providing the first demonstration that *Drosophila* can produce phenotypes related to human disease [1]. Genome sequencing has enabled more systematic identification of *Drosophila* orthologues of human disease genes. Initial analysis of the fly genome [2] identified 289 orthologues of disease genes and estimated that over 60% of human disease genes are well conserved in the fly. Today, over 800 human disease model reports, detailed descriptions of links between specific human diseases and *Drosophila* genes, are available on FlyBase (FB2018_03) [3].

With the advent of low-cost next-generation sequencing and improved mechanisms for data sharing, the pace of discovery of human genes linked to genetic disease has increased dramatically in the last several years. Approximately 1500 new human rare disease genes have been discovered since the start of the present decade [4,5]. While some of these genes are components of well-established pathways, functional information for many others is limited. Studies to obtain such information in humans can be difficult when the affected population for a given disease is very small and when the phenotype is complex and/or variable.

Model organisms such as *Drosophila* are extremely valuable to the study of human rare diseases [6,7]. Often, fundamental cell and developmental biology research in model organisms has produced a body of knowledge about genes before their human counterparts have been linked to any clinical phenotype [8]. Thus, when such a link is made, insights gained previously from work in the fly can inform how to address the role of the orthologous human gene in disease. Additionally, model organisms can be used to help confirm whether or not a particular candidate genetic variant is causative for the disease. With CRISPR-Cas9 technology it is usually straightforward to produce the corresponding genetic variant in flies and determine whether a phenotype related to the human disease is produced [9,10]. *Drosophila* also provides the most efficient system for genome-wide genetic screens for modifier loci, thanks to comprehensive collections of publicly-available fly lines that express short interfering RNA (shRNA) under upstream activator element (UAS)-mediated control that target essentially all genes [11,12,13], as well as large public collections of GAL4 lines that can drive shRNA expression in a wide range of cell and tissue types [14,15]. Such a screen has, for example, been successfully used to find *SMARCB1* interacting genes relevant to atypical teratoid/rhabdoid tumors [16].

The International Rare Diseases Research Consortium (IRDiRC), which brings together public and private funders, and patient organizations to coordinate and better facilitate rare diseases research, has recognized the importance of model organisms to rare diseases research. Among IRDiRC’s explicit policies is the promotion of “coordination between human and model systems research in rare diseases” [17]. Consistent with this, several agencies have invested in specific measures to fund research using model organisms to investigate questions relevant to human rare disease. In this article we will review these initiatives in order to help make them better known in the *Drosophila* research community and to demonstrate the value of taking such an approach.

## 2. Rare Diseases: Models & Mechanisms (RDMM) Network

The Canadian Rare Diseases: Models & Mechanisms (RDMM) Network [18] establishes collaborations between clinicians discovering rare disease genes and researchers who can analyze equivalent genes and pathways in model organisms. The goal of the RDMM is to generate new biological and therapeutic insights with the intent to improve the lives of those affected by rare diseases. The RDMM is funded by the Institute of Genetics of the Canadian Institutes of Health Research, Genome Canada, and Genome British Columbia, and its administration is provided by the Maternal Infant Child and Youth Research Network (MICYRN) [19].

**Figure 1:**
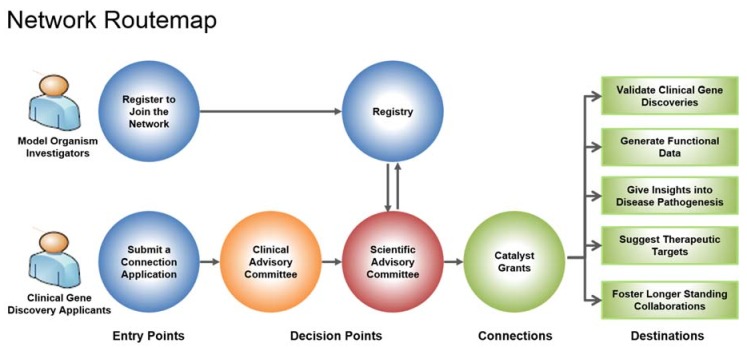
RDMM Network Routemap. Clinicians that have discovered a new disease gene submit a connection application to the Clinical Advisory Committee (CAC). If approved, the gene goes to the Bioinformatics Core (BIC) to conduct a search in the network registry to find model organism researchers working on the orthologue of the disease gene. The Scientific Advisory Committee (SAC) will then invite researchers to submit a two-page application to be reviewed. If approved, the grant (CAD 25,000) is awarded to fund these collaborative experiments. RDMM-Rare Diseases: Models & Mechanisms

Genes identified as being associated with a rare disease are submitted by a clinician through a connection application in the hopes of connecting to a model organism researcher (Figure 1). The connection application is evaluated by the Clinical Advisory Committee (CAC) and if approved, the Bioinformatics Core (BIC) will do a search in the network registry to find matches of model organism researchers working on the gene of interest. The network registry is comprised of researchers across Canada working on various model organisms, such as *Caenorhabditis elegans*, *Drosophila*, mouse, yeast and zebrafish. To date, 483 researchers have registered with the network. Model organism researchers found to be potential matches are then invited to submit a catalyst grant proposal to study the gene. The proposal is reviewed by the Scientific Advisory Committee (SAC) and when a suitable match is identified between clinician and researcher, a catalyst grant of CAD 25,000 is awarded.

Thus far, the RDMM has awarded 71 catalyst grants. Nine of these grants have supported studies in *Drosophila*, and one of these has resulted in a research publication. This study looked at mutations in the *TMTC3* gene found to be associated with periventricular nodular heterotopias (PVNHs), which are among the most common brain malformations in humans. They result from defects in neuronal migration during early brain development [20], and affected individuals often present with nocturnal seizures and intellectual disability.

Four affected siblings in an affected family were all found to be compound heterozygotes of two variants in *TMTC3*; one that produces a missense mutation (R71H) and another that produces a frameshift that truncates the normally 914-amino-acid protein after amino acid 728 [21]. *TMTC3* contains nine tetratricopeptide (TPR) repeats and the frameshift mutation results in the loss of the final three of these. R71 is not located within a known functional domain but is conserved in mammalian, zebrafish, and *Drosophila* orthologues. This suggested that analysis of model organisms deficient for the counterpart gene might help validate the link between *TMTC3* and the observed phenotypes, and provide more functional information about the gene.

Little had been previously published on the *Drosophila TMTC3* orthologue *CG4050*. To investigate a potential role for in seizure biology, its activity was specifically knocked down in neurons using a UAS-responsive RNAi expressing transgenic line and a neuron-specific GAL4 driver (*elav*-*GAL4*). A paradigm for seizures, called the bang sensitivity assay, has been established in *Drosophila* [22]. In this assay, flies are mechanically disturbed by subjecting their culture tube to a vortex mixer, and afterward the time required for flies to right themselves on their feet is measured. As assessed in this way, flies with reduced neuronal *CG4050* expression showed an increased susceptibility to mechanically induced seizures. Importantly, this phenotype was rescued by expression of a human *TMTC3* transgene in post-mitotic neurons, again using *elav*-*GAL4*, confirming that loss of *CG4050* indeed leads to seizure susceptibility and supporting a role for *TMTC3* in the nocturnal seizures seen in patients.

## 3. UDN and Centers for Mendelian Genomics

The US National Institutes of Health (NIH) have recognized rare and undiagnosed diseases as a research priority. In 2008, the National Human Genome Research Institute (NHGRI) and the Office of Rare Diseases Research (ORDR) launched an internal initiative funded through the NIH Common Fund called the Undiagnosed Diseases Program (UDP), through which individuals whose conditions have eluded diagnosis may apply to undergo a week of diagnostic tests at the NIH Clinical Center. This program identified a large unmet need, in that resources only allowed fewer than 25% of accepted applicants to be seen. It was therefore extended in 2012 into a network of seven clinical sites and renamed the Undiagnosed Diseases Network (UDN) [23], which has in turn entered a second phase of funding.

At present, the UDN is generating approximately 200 gene variants per year that are strong candidates for being causative in patients with unknown disorders. Recognizing the value of model organisms for testing the pathogenicity of genetic variants found in human patients, the NIH Common Fund launched a request for applications for a Model Organism Screening Center (MOSC) to be part of the UDN. It was required that the center would include *Drosophila* and zebrafish as model systems. An award was subsequently made, and sites were established at the Baylor College of Medicine in Houston for *Drosophila*, as well as the University of Oregon for zebrafish. The *Drosophila* group had previously published a landmark paper reporting their production of a large bank of X-linked mutations affecting neuronal maintenance, using them as a resource to study human Mendelian disease genes [24,25]. A competition is presently underway for a second phase of funding for the MOSC that would sustain it until 2022.

The MOSC responds to receipts of genetic variants and phenotypic information about patients seen at UDN clinical sites that have not been diagnosed. It has developed a software tool called MARRVEL (Model Organism Aggregated Resources for Rare Variant Exploration), which aggregates existing information about the human genetic variant and about model organism orthologues of the gene it affects [26]. When a variant from this analysis emerges as a high priority, an experimental program commences in either the fly or the zebrafish core groups. MARRVEL, which is publicly available [27], promises to make it far easier to use public genetic resources to prioritize rare human genetic variants for study in model organisms, which could lead to many additional collaborations between clinical and model organism geneticists outside of the UDN.

Several papers have now emerged from MOSC, validating the overall approach. One such paper uses *Drosophila* to validate genetic variants in *EBF3*, which encodes the transcription factor Early B Cell Factor 3, as causative for a neurodevelopmental disorder involving global developmental delay, intellectual disability, speech disorder, and malformations of the central nervous system and genitourinary system [28]. Three unrelated individuals with these phenotypic characteristics were found to carry de novo missense mutations in *EBF3*, either R163Q or R163L, affecting an amino acid that resides within a conserved zinc-finger domain of the protein. Mammals have four genes that produce proteins closely related to *EBF3*, but *Drosophila* has only one, called *knot* (*kn*).

The gene *kn* has been extensively studied and is linked to many developmental processes, including dendrite morphogenesis, at least in part through activating expression of *Ten-m*, a gene encoding a cell adhesion molecule [29]. Homozygous null mutants of *kn* die during embryonic development [30]. Transgenic flies were produced that expressed either the human wild-type *EBF3*, or the R163Q or R163L mutant forms, under the control of the *Drosophila kn* promoter. When these transgenes were introduced into a *kn* null mutant background the wild-type *EBF3*, but neither mutant form, could rescue lethality and produce viable adult flies. This experiment helped to validate the pathogenicity of these two mutant alleles.

Another paper involving the MOSC that used *Drosophila* implicates a different transcriptional regulator, *TBX2*, in a syndromic cardiovascular and skeletal developmental disorder that is related to DiGeorge syndrome [31]. While *TBX2* has been studied extensively in mice and rats—where it is an important developmental regulator in heart, brain, eye, bone, and limbs [32,33,34,35,36]—surprisingly it had not been heretofore clearly linked to a human Mendelian disorder. Four affected individuals from two unrelated families were determined to be heterozygotes for missense mutations in *TBX2*, producing at the amino acid level either R20Q or R305H. Phenotypically, these individuals showed congenital cardiac defects, skeletal abnormalities, facial dysmorphia, developmental delay, and endocrine system disorders.

The *Drosophila* orthologue of *TBX2* is called *bifid*, and it is essential for viability [37]. Attempts to rescue *bifid* mutants with even a *Drosophila* wild-type transgene were unsuccessful, presumably because of extreme dosage sensitivity. However, since the human disease is inherited as a dominant allele, experiments investigating the effects of ectopic expression in flies in a wild-type genetic background could be conducted. Expression of either wild-type *bifid* or human *TBX2* in eyes using the *eyeless–Gal4* (*ey–Gal4*) driver produced over 50% lethality and severely reduced the eye size in 63–71% of the flies that eclosed. Conversely, fewer than 20% of eclosed flies showed severe eye size reduction when either the R20Q or R305H variant of *TBX2* was expressed, and for R305H, viability was also substantially greater. These constructs were also expressed in adult photoreceptor cells using the *Rhodopsin1–Gal4* driver, and photoreceptor activity was measured using electroretinograms. While overexpression of the fly and human wild-type forms produced severe defects in phototransduction, much less severe effects were obtained from either mutant form. Taken together, these results indicated that both missense alleles cause a reduction of function of the *TBX2* protein.

Another paper using *Drosophila* and involving the MOSC linked *ATP5F1D*, which encodes a subunit of mitochondrial ATP synthase, to a human mitochondrial disease [38]. Two patients were identified with missense mutations causing substitutions in amino acids (P82L and V106G) that are conserved between humans and flies. The effects of these mutations were analyzed by expressing transgenes carrying them in flies and assessing their ability to rescue lethality induced by expression of siRNA targeting *ATPsyn*, the homologue of *ATP5F1D*. While wild-type *ATP5F1D* was able to rescue, neither mutant form could do so. When siRNA targeting *ATPsyn* is expressed with *ey–Gal4*, most of the head is lost; this phenotype is also rescued by wild-type *ATP5F1D*, but only partially so with either mutant form. These results confirm the pathogenicity of the two mutant alleles.

In another recent publication, Luo and UDN colleagues expressed two rare human variants of *CACNA1A*, which encodes a neuronal calcium channel subunit, in *Drosophila* and showed that they have different phenotypic effects, consistent with the clinical symptoms of the patients carrying the different alleles [39]. *CACNA1A* mutations have been linked to several neurological disorders in humans, including spinocerebellar ataxia type 6, episodic ataxia type 2, and familial hemiplegic migraine [40]. Missense mutations leading to the amino acid substitutions R1673P or R1664Q in *CACNA1A* were identified in five patients with global developmental delay and congenital ataxia. These particular variants had not heretofore been studied, and as both arginine residues are conserved in *Drosophila*, the fly could be used to study their effects on gene function. Mutants for the fly homologue *cac* are recessive lethal, and in mosaic eye clones *cac* mutations lead to aberrant lysosome–autophagosome fusion, expanded nerve terminals, and excessive accumulation of synaptic vesicles [41]. Wild-type and mutant forms of *Drosophila cac* were tested for their ability to rescue lethality; while the wild-type form could do so, R1673P completely failed to rescue, and R1664Q did so only partially. Electroretinograms recorded from *cac* mutant eyes showed loss of the ‘on’ and ‘off’ transients. This phenotype was also observed in eyes expressing the R1664Q form, but expression of R1673P produced an opposite phenotype, drastically increasing the amplitude of those transients. Analysis of eye structure by transmission electron microscopy further showed that R1664Q resembled the *cac* mutant, while expression of R1673P produced a much more dramatic degeneration phenotype. Remarkably, the human patient with the R1673P allele had cerebellar degeneration, but the other four patients with the R1664Q allele did not.

Finally, a recent report from the UDN and MOSC has linked loss-of-function truncation mutations in the Interferon Regulatory Factor 2 Binding Protein Like (*IRF2BPL*) gene with a syndrome involving severe neurodevelopmental regression, hypotonia, ataxia, seizures, and lack of coordination [42]. Null mutations in the *Drosophila* homologue *pits* (Protein interacting with Ttk69 and Sin3A) are lethal, while partial knockdown of *pits* by RNA interference produces neurodegenerative phenotypes. The truncation mutations found in the human patients behave as severe loss-of-function alleles when produced in flies.

Researchers affiliated with the MOSC also collaborate with Centers for Mendelian Genomics (CMG) to help identify new genes linked to human rare diseases. One paper resulting from such a collaboration [43] built upon knowledge about *Drosophila* Ariadne-1 (Ari-1), which is a component of the Linker of Nucleoskeleton and Cytoskeleton (LINC) complex that is involved in linking nuclei to the cytoskeleton [44]. Ari-1 is also an E3 ubiquitin ligase of the RING-between-RING (RBR) type, and is closely related in structure to Parkin, linked in humans to early-onset Parkinson’s disease [45]. Mutants in *ari-1* in flies are recessive lethal at the late pupal stage, exhibiting nuclear clustering and aberrant larval muscle morphology, and analysis of genetic mosaics indicated additional roles for *ari-1* in synaptic transmission and sensory bristle development [43]. The lethality of *ari-1* could be rescued by ubiquitous expression of its human homologue, *ARIH1*, indicating functional conservation between *Drosophila* and humans. Further experiments demonstrated that Ari-1 ubiquitinates Klaroid (Koi), a component of the LINC complex, regulating its levels by targeting it for proteolytic degradation.

With the help of MARRVEL and GeneMatcher [46], which enables querying of large patient datasets for particular genetic variants, three human patients were discovered with variants in *ARIH1*; one with a premature stop codon (R171*) and two missense mutations causing the amino acid substitutions E15Q and E44G. These patients have thoracic aortic aneurysms and dysfunction of smooth muscle cells [43]. Unlike wild-type human *ARIH1*, all three mutant forms failed to rescue nuclear positioning and lethality when expressed in flies, confirming the pathogenic nature of these mutations. Based on the phenotypes observed in flies, nuclear morphology was examined in vascular smooth muscle cells from patient samples, and irregular nuclear envelopes with unusual folds and indentations were observed. These results confirm that Ari-1 functions in regulating nuclear morphology in both flies and humans and is linked to genetic disease.

In another collaboration between MOSC and CMG, *Drosophila* was used to identify *ATAD3A*, a nuclear gene that encodes a mitochondrial membrane-bound AAA-domain ATPase, as a causative gene for a heritable syndrome that involves developmental delay, hypotonia, optic atrophy, axonal neuropathy, and hypertrophic cardiomyopathy [47]. One missense allele of *ATAD3A* found in some of these patients produced the amino acid substitution R528W. Expression of the corresponding mutation (R534W) in the *Drosophila ATAD3A* homolog, *belphegor* (*bor*) [48], in all neurons or in motor neurons produced lethality while expression of the wild-type version caused no detectable phenotype. In tissues expressing *bor*^R534W^, the signal intensity for ATP5A, a mitochondrial marker, was greatly reduced, and analysis by transmission electron microscopy showed aberrant mitochondrial morphology and evidence of extensive mitophagy.

A third collaborative project between a CMG and MOSC *Drosophila* researchers has led to a link between Nardilysin, a mitochondrial co-chaperone protein involved in folding of the Krebs cycle enzyme-ketoglutarate dehydrogenase, and a patient registered on GeneMatcher with a syndrome involving severe global developmental delay and ataxia [49].

These several reports from the RDMM and MOSC produce compelling evidence of the value of *Drosophila* to validate the pathogenicity of human genetic variants and to provide insight into the functional consequences of such variants.

## 4. Solve-RD

The European Commission (EC) has been dedicating substantial funds to rare disease related research for a number of years. One of its latest initiatives is the investment of more than EUR 15M from the EC’s Horizon 2020 program that was announced in January 2018 for Solve-RD [50], a large consortium led by the University of Tübingen in Germany, the Radboud University Medical Center in Nijmegen, the Netherlands, and the University of Leicester in the UK. The overall goal of the consortium is to improve diagnosis of rare diseases by sharing of genomic data among participating centers, and by using further high-throughput methods such as transcriptomics, proteomics, metabolomics, and epigenomics to develop diagnostics for rare diseases for which single causative genes have not yet been identified through standard short-read DNA sequencing approaches. To do this the consortium plans to re-analyze 19,000 unsolved patients from European Reference Networks who have already undergone exome sequencing. An important part of this effort is to implement a system related to the RDMM in which experts in model organisms will be linked to clinical geneticists with novel candidate loci to test the pathogenicity of genetic variants and to provide information on genetic pathways linked for the first time to a disease.

## 5. Japanese RDMM

The Initiative on Rare and Undiagnosed Diseases (IRUD) of the Japan Agency for Medical Research and Development (AMED) has included an initiative patterned on the RDMM as part of its IRUD Beyond set of projects launched in November 2017. It is intended that the Japanese RDMM will soon be opened to non-Japanese researchers, and an English-language website intended to accomplish this went live in July 2018 [51].

## 6. Concluding Thoughts

A growing body of literature proving the value of targeted efforts to link human geneticists working on rare diseases with *Drosophila* researchers is emerging. This will only magnify as existing efforts mature and new efforts related to those described here, such as Solve-RD and others that are in the discussion stage, come on track. To further increase the reach of these initiatives, there are plans to extend networks such as RDMM to enable them to transcend national boundaries, so that a clinical geneticist in one participating jurisdiction could establish a collaboration with a *Drosophila* researcher in a different one.

MOSC and the RDMM represent two different forms of a collaborative network, in that MOSC has established a single model organism center that is tightly integrated with clinical geneticists in the UDN, while RDMM uses a ‘matchmaking’ service to incentivize collaborations between a clinical geneticist and anyone with appropriate expertise in the broadly distributed model organism genetics community. It will be important to see which of these models proves to be more effective, and whether the more centralized model can evolve in a way that enables participation by fly researchers and clinical geneticists who are not affiliated with the center.
